# Neonatal Hypoglycaemia Management Guideline appraisal using the AGREE II instrument and report of variations in unit guidelines in Australia and New Zealand

**DOI:** 10.1111/jpc.16729

**Published:** 2024-12-02

**Authors:** David Thomas Mc Hugh, Rosalynn Pszczola, Joanne M Said

**Affiliations:** ^1^ Monash University Melbourne Victoria Australia; ^2^ Cardiff University Cardiff United Kingdom; ^3^ Monash Health Melbourne Victoria Australia; ^4^ Royal Children's Hospital Melbourne Victoria Australia; ^5^ Maternal Fetal Medicine Joan Kirner Women's and Children's, Sunshine Hospital, Western Health Melbourne Victoria Australia; ^6^ Department of Obstetrics, Gynaecology and Newborn Medicine University of Melbourne Melbourne Victoria Australia

**Keywords:** AGREE II, blood glucose, diagnosis, hypoglycaemia, neonatal screening, infant, newborn

## Abstract

**Aim:**

To assess the quality and rigour of Neonatal Hypoglycaemia guidelines used in the major Australian and New Zealand neonatal care centres. To compare and highlight any major differences in management guidelines between centres.

**Methods:**

All level III NICUs in Australia and New Zealand were invited to participate. The AGREE II (Appraisal of Guidelines, Research & Evaluation) was used to critically appraise the guideline for the management of neonatal hypoglycaemia. Recommendations regarding definition, treatment, method of testing and admission criteria were compared from the guidelines provided.

**Results:**

Neonatal Hypoglycaemia guidelines were received from 19 of the 29 invited hospitals; two guidelines were excluded as the hospitals providing these guidelines did not provide care for inborn neonates. None of the 17 guidelines received a standardised score of 50% or higher on all six domains of the AGREE II tool. The mean scores of each of the AGREE II domains were as follows: Scope and Purpose 76%; Stakeholder Involvement 41%; Rigour of Development 20%; Clarity of Presentation 66%; Applicability 30% and Editorial Independence 0.1%. The glycaemic threshold defining hypoglycaemia varied between 2.0 and 2.6 mmol/L in the guidelines. True blood glucose using either a glucose oxidase method or blood gas analyser was recommended as the first line test in 35% of the guidelines. Fifteen of the 17 guidelines recommended buccal gel as first‐line treatment of hypoglycaemia.

**Conclusions:**

Neonatal Hypoglycaemia guidelines are of varying methodological quality. There are inconsistences in the management of hypoglycaemia across neonatal units in Australia and New Zealand.

## What is already known on this topic

1. Hypoglycaemia is amongst the most common neonatal conditions, affecting 15% of all births. There is a rise in incidence year‐on‐year due to changing maternal population factors.
2. The lack of consistency in Neonatal Hypoglycaemia Management Guidelines was an observation that had been made in the preparation of the C*STEROID and PRECeDe obstetric trials which require neonatal monitoring for hypoglycaemia and appropriate management.
3. Previous evidence has shown variance in recommendations in the management of neonatal hypoglycaemia.

## What this paper adds

1. There is varying quality and rigour of development of guidelines which may lead to the observed inconsistences.
2. Recommended thresholds for diagnosis, treatment and neonatal nursery admission all varied widely across different guidelines of level III NICUs in Australia and New Zealand.
3. This study further provides evidence for a unifying bi‐national guideline between Australia and New Zealand.

Hypoglycaemia is amongst the most common neonatal conditions affecting 15% of all births. That incidence rises to almost 50% in those with identified risk factors who undergo screening.^1^, ^2^ There is a rise in incidence year‐on‐year due to changing maternal population factors.^3^

The association between severe, prolonged hypoglycaemia in the neonatal period and subsequent neurodevelopmental impairment was first described 60 years ago. Since then debate has continued about the detection, diagnosis and optimal management of neonatal hypoglycaemia.^4^, ^5^ A treatment threshold of blood glucose concentration <2.6 mmol/L has been widely agreed and used since the 1980s.^6^ Recent research, however, has suggested that this may no longer be accurate with blood glucose concentrations of <1.4 mmol/L only needing treatment in the first 4 h of life and above 2.0 mmol/L being both acceptable and safe up to 24 h of age.^7^ High blood glucose concentrations during recovery from hypoglycaemia^6^ also have detrimental developmental outcomes^8^ indicating a need to carefully select the group of neonates who warrant screening and review of the thresholds for intervention. A significant change to neonatal hypoglycaemia management in the last decade has been the use of buccal dextrose gel as an adjunct to early and regular feeds.^8^

Despite the debate, inconsistency and lack of high‐quality evidence, management guidelines for neonatal hypoglycaemia exist in almost all centres caring for newborns. These address the importance of identifying neonates deemed at risk and make recommendations regarding the need for and timing of blood glucose monitoring, interventions aimed at preventing hypoglycaemia, the threshold for treatment and what that treatment should be. The balance between minimising risk of future neurodevelopmental impairment, limiting painful interventions and avoidance of separating mother and baby whilst promoting breastfeeding are important overriding principles for these guidelines. Rajay and Harding^9^ recently reported significant variation in guidelines for managing neonatal hypoglycaemia in centres participating in a clinical trial of neonatal hypoglycaemic management.

The use of local practice guidelines aims to assist practitioners to improve consistent care provision, resource utilisation and decision making with reference to details specific to their organisation.^10^ Australia lacks a funded national body to provide overarching guidance as exists in the UK, America and Canada and so health services and hospitals develop their own guidelines with periodic review.^4^ Properly developed, guidelines promote evidence based practice and discontinuation of outdated practices.^11^ However, potential risk exists when non‐rigorous methodology is applied to guideline development that care provided is not patient‐centred or applied to inappropriate patient populations. Higher quality guidelines developed with rigorous and transparent methodology reflect the perspective of stakeholders, are based on up‐to‐date evidence, free from any conflict of interest, have undergone critical expert review and are specific and non‐ambiguous.^12^

The lack of consistency and conformity in Neonatal Hypoglycaemia Management Guidelines was an observation that had been made by our team whilst preparing a large obstetric randomised controlled trial^13^ and so we aimed to quantify these differences and assess the clinical practice guidelines currently in use in Australian and New Zealand tertiary neonatal hospitals for adherence to evidence base and overall quality.

## Aims

To assess the quality and rigour of Neonatal Hypoglycaemia guidelines used in the major Australian and New Zealand neonatal care centres using a recognised and validated tool.

To compare and highlight any major differences in management guidelines between centres.

## Methods

All 29 tertiary perinatal neonatal units within Australia and New Zealand were identified from the Australia New Zealand Neonatal Network Annual report and each director was contacted *via* email to explain the study, provide a copy of the ethics approval letter, and invite their participation by providing a copy of the guidelines used within their unit to manage neonates at risk of hypoglycaemia soon after birth. If differing guidelines for management of hypoglycaemia in different clinical circumstances existed, only those that applied to otherwise well newborns being managed on the postnatal ward were included in the analysis. If no reply was received within 2 weeks, a reminder email was sent. If a positive response was received and the guidelines provided, then consent to participate was assumed. Guidelines were deidentified for the purpose of this analysis.

AGREE II (Appraisal of Guidelines, Research & Evaluation) is an instrument developed for the structured review and assessment of practice guidelines.^14^, ^15^ The AGREE Collaboration is comprised of international guideline developers and researchers who published the initial AGREE tool in 2003 with revision and redesign leading to the release of its second version in 2010. The AGREE II instrument rates 23 items over six domains with quality ratings from 1 (lowest) to 7 (exceptional) for each item. A final subjective numerical score out of 7 is also given to the guideline in its entirety with the option to recommend the guideline either for use, for use with modifications or not recommend. Item and domain scores were calculated by scaling as a percentage of the total possible score out of 100%. The AGREE II's reliability and validity is well established^14^ and it has been used previously to assess neonatal guidelines.^16^, ^17^

Analysis was undertaken by two assessors trained in the use of AGREE II (https://www.agreetrust.org).^14^ If there was a difference in each partial score (≥2) we planned to undertake further assessment by a third assessor to reach consensus, however this circumstance did not eventuate.

## Results

### Guideline characteristics

Neonatal Hypoglycaemia guidelines were received from 19 of the 29 invited hospitals from Australia (n = 13) and New Zealand (n = 6). Of the 10 hospitals that did not respond were 9 were level III neonatal units based in Australia, one hospital was a Paediatric Intensive care unit and so did not have a neonatal hypoglycaemia guideline. We subsequently excluded two guidelines as they were from tertiary referral units that only provided care for outborn neonates. This left a total of 17 guidelines for analysis.

### AGREE II appraisal

None of the 17 guidelines received a standardised score of 50% or higher on all six domains. The mean overall score for each domain of the 17 included guidelines are presented in Table 1.

**Table 1:** Agree II domain scores for each guideline; mean (%)

Guideline number	Year of guideline publication	1: Scope and purpose	2: Stakeholder involvement	3: Rigour of development	4: Clarity of presentation	5: Applicability	6: Editorial independence	Overall assessment
1.	May, 2019	91	75	34	80	54	1	83
2.	May, 2022	86	67	34	78	47	1	91
3.	Oct, 2022	55	41	10	56	23	0	66
4.	Nov, 2021	86	36	25	67	44	0	83
5.	Feb, 2022	33	67	15	81	8	0	83
6.	May, 2022	81	31	16	69	17	0	75
7.	Feb, 2021	91	11	1	72	25	0	66
8.	Sep, 2020	94	69	26	72	35	0	75
9.	May, 2016	50	33	13	13	25	0	83
10.	Dec, 2021	100	69	26	72	35	0	83
11.	Apr, 2022	89	47	25	100	43	0	83
12.	Dec, 2019	72	22	24	94	42	0	83
13.	Sep, 2019	25	14	8	72	8	0	41
14.	July, 2020	83	25	33	74	25	0	66
15.	Apr, 2020	89	27	18	44	10	0	41
16.	July, 2022	69	39	11	50	38	0	58
17.	Nov, 2020	92	30	25	33	42	0	75

#### Domain 1: Scope and purpose

This relates to if the overall objectives and population the guideline refers to are specifically described.^14^ The average score of the guidelines for this domain was 76% with most of the guidelines describing the overall objectives, health question and target population (Table 2). Individual scores for each guideline in this domain are described in Table 1.

**Table 2:** Pooled results; mean, minimum and maximum standardised scores (%) for the items in each domain of the AGREE II tool

Domain	Item	Mean (%)	Minimum (%)	Maximum (%)
1. Scope and purpose	1: Objectives	71	8	100
	2: Health question	66	16	100
	3: Target group	76	25	100
2. Stakeholder involvement	4: Group membership	32	0	100
	5: Target population preferences	26	0	100
	6: Target users	73	16	100
3. Rigours of development	7: Search methods	17	0	33
	8: Evidence selection	15	0	42
	9: Evidence quality	29	0	58
	10: Recommendations linked to evidence	18	0	33
	11: Benefits and harms	42	0	67
	12: Link to evidence	34	0	75
	13: External review	16	0	50
	14: Procedure for update	24	0	75
4. Clarity of presentation	15: Unambiguous recommendations	72	58	100
	16: Management options	56	8	100
	17: Identifiable recommendations	67	25	100
5. Applicability	18: Facilitators and barriers	44	0	83
	19: Implementation advice	46	16	92
	20: Resource implications	30	0	58
	21: Auditing criteria	19	0	67
6. Editorial independence	22: Funding body	0.01	0	8
	23: Competing interests	0.0	0	0

#### Domain 2: Stakeholder involvement

This relates to whether guidelines were developed with individuals from relevant professional craft groups and subspecialties, if views of the target population were sought and if the target users of the guidelines are clearly defined.^14^ The mean score for this domain was 41%. Only one guideline reported that a consumer representative was involved in the guideline development. The majority of guidelines, 65% (n = 12) stated that stakeholders were consulted in the writing of the guideline.

#### Domain 3: Rigour of development

This is the largest domain in the AGREE II and relates to the methods used to collect evidence to formulate the guideline.^14^ This was the second lowest scoring domain with an average score of 20% (Table 2). Most guidelines had a large number of references but failed to describe any strategies used to search, review and appraise the evidence.

#### Domain 4: Clarity of presentation

This refers to how specific, unambiguous and easily identifiable recommendations were.^14^ This was the second highest scoring domain (66%) and 13 of the guidelines scoring >50%.

#### Domain 5: Applicability

This relates to how the recommendations can be put into practice and how the guideline considers the resource implications of its use. This was one of the lowest scoring domains with the average score being 30% and only 1 of the guidelines scoring greater that 50%.

#### Domain 6: Editorial independence

This refers to ensuring the guideline development was free from bias. None of the guidelines mentioned competing interests or addressed the influence of author affiliations.

#### Overall assessment

This score was made by the assessors as an overall impression of guideline and its use. The mean score for this domain was 75%.

### Recommendations from guidelines

This assessment was made by the assessors if the guidelines could be recommended for use. Eight of the guidelines were recommended for use, seven were recommended with changes and two were not recommended for use.

#### Hypoglycaemia definition and method of testing

This study has identified sufficient evidence to reignite the debate on what is an acceptable blood glucose concentration early in life. Neonatal hypoglycaemia was defined as a blood glucose concentration of <2.6 mmol/L in 12 out of the 17 guidelines. Two guidelines used the threshold of <2.0 mmol/L in the first 24 h, and one other guideline used a threshold of <2.0 mmol/L in the first 4 h, if clinical signs of hypoglycaemia are absent, and <2.6 mmol/L after 4 h. One outlier guideline used a definition of hypoglycaemia of <2.6 mmol/L in first 48 h and <3.3 mmol/L thereafter.

#### First treatment

One guideline recommended intravenous glucose as the first method of treatment and did not recommend buccal glucose gel or supplementary feeding. One guideline recommended supplementary formula feeding or intravenous glucose for an episode of hypoglycaemia, only recommending buccal or nasogastric glucose gel if there was a delay in commencement of intravenous glucose or caregivers did not consent to formula feeding. The other 15 guidelines recommended buccal glucose gel as first line treatment for hypoglycaemia.

Differences in threshold for treatment of hypoglycaemia also existed. Two guidelines defined hypoglycaemia as <2.0 mmol, however, if the neonate was asymptomatic and blood glucose concentration was between 2.0 and 2.6, they recommended 3 hourly frequent feeding rather than treatment with buccal gel.

All 17 guidelines recommended checking blood glucose level 30 min after an intervention for hypoglycaemia was implemented. Eleven guidelines required three blood glucose levels >2.6 mmol/L to stop monitoring. One guideline recommended two blood glucose levels >2.0 at 1 and 4 h, however, did not discuss any monitoring beyond this. Four guidelines recommended a minimum of 24 h of blood glucose concentration monitoring for neonates at risk or who had a hypoglycemic episode.

#### Admission criteria

The threshold for admission to the nursery amongst guidelines was based on glucose threshold and failed enteral or buccal treatment. The criteria for admission and escalation of treatment varied significantly. All were based on a combination of low blood glucose, and response to initial treatment. The most common admission threshold was ‘Any blood glucose concentration <1.5 mmol/L’ or ‘blood glucose <2.6 mmol/L after two doses of glucose gel’ with 7 of 17 units using this threshold. From 17 guidelines, there were 9 differing admission thresholds.

## Discussion

Critical appraisal of neonatal hypoglycaemia guidelines currently in use in Australia and New Zealand has revealed guidelines to be of varying quality and adherence to evidence. We found that ‘Rigor of Development’ in the AGREE II tool to be one of the lowest scoring domains.

There is variation in recommendations between guidelines used throughout Australia and New Zealand. This may lead to inconsistencies in delivery of treatment amongst staff who move between hospitals.

One guideline recommended treatment for neonates with intravenous glucose as the primary management of hypoglycaemia which potentially may lead to increased admissions to the neonatal nursery (contributing to increased health‐care costs) and separation of neonates from their mothers which may in turn delay lactation and exacerbate or prolong neonatal hypoglycaemia.^18^

Further research is required not only on the treatment threshold of neonatal hypoglycaemia but also on the gold standard treatment regimen. The variance between guidelines may be due to the selection bias of studies. Guidelines that use a lower blood glucose threshold referenced studies with results that found no difference between neurodevelopmental outcomes between lower threshold levels versus the higher traditional level of 2.6 mmol/L.^19^ Guidelines that recommended higher treatment threshold of 2.6 mmol/L referenced studies that supported this management.^20^

Many of the guidelines failed to seek consumers'/parents' views and preferences. Only one guideline reported using a consumer parent group as part of the review process. It is important that parents are involved with guideline development to promote family‐centered care.^21^

### Limitations

Many guidelines did not report the methodology used to generate literature search criteria, appraisal techniques and ‘Strengths and weaknesses of evidence’ and so accurate appraisal of this domain could not be undertaken. Whilst it may be reasonable to assume that this stage of guideline development was performed well and this information omitted to achieve brevity and improve clarity, there is no way of assessing if this is indeed the case. It is notable that AGREE II was developed in the United States and funding bodies or conflicts of interest are much less relevant within Australasia where the majority of health care is publicly funded, this domain was included in the appraisal for completeness only.

The AGREE II tool lacked guidance about how to score overall assessment or on how the ‘final recommendations for use item’ should be calculated.

### Future implications

A solution to the variation in the guidelines would be a bi‐national unified guideline between states and units. This has been achieved in Australia and New Zealand previously with the establishment of a unified parenteral nutrition guideline.^22^ This consensus guideline should be based on high quality evidence and state the definition of hypoglycaemia, agreement on risk factors for screening, recommendations for hypoglycaemia management and threshold for neonatal nursery admission.
